# Critical amino acids for the insecticidal activity of Vip3Af from *Bacillus thuringiensis*: Inference on structural aspects

**DOI:** 10.1038/s41598-018-25346-3

**Published:** 2018-05-15

**Authors:** N. Banyuls, C. S. Hernández-Rodríguez, J. Van Rie, J. Ferré

**Affiliations:** 1 ERI de Biotecnología y Biomedicina (BIOTECMED), Departamento de Genética, Universitat de València, 46100 Burjassot, Spain; 2 Bayer CropScience N.V., Ghent, Belgium

## Abstract

Vip3 vegetative insecticidal proteins from *Bacillus thuringiensis* are an important tool for crop protection against caterpillar pests in IPM strategies. While there is wide consensus on their general mode of action, the details of their mode of action are not completely elucidated and their structure remains unknown. In this work the alanine scanning technique was performed on 558 out of the total of 788 amino acids of the Vip3Af1 protein. From the 558 residue substitutions, 19 impaired protein expression and other 19 substitutions severely compromised the insecticidal activity against *Spodoptera frugiperda*. The latter 19 substitutions mainly clustered in two regions of the protein sequence (amino acids 167–272 and amino acids 689–741). Most of these substitutions also decreased the activity to *Agrotis segetum*. The characterisation of the sensitivity to proteases of the mutant proteins displaying decreased insecticidal activity revealed 6 different band patterns as evaluated by SDS-PAGE. The study of the intrinsic fluorescence of most selected mutants revealed only slight shifts in the emission peak, likely indicating only minor changes in the tertiary structure. An *in silico* modelled 3D structure of Vip3Af1 is proposed for the first time.

## Introduction

Vegetative insecticidal proteins (Vip) are entomopathogenic proteins of increasing importance in the framework of sustainable pest management and crop protection strategies. Different to the well-known δ-endotoxins, Vip proteins are produced during the vegetative growth phase of *Bacillus thuringiensis* (Berliner) (Bt) and secreted to the growth medium as soluble proteins. Of particular interest are Vip3A proteins, which were first described in the late 90’s and were found to be active against lepidopteran species with potencies different to those of the widely used Cry proteins, such as the Cry-tolerant *Agrotis ipsilon* (Lepidoptera: Noctuidae) or of many other species from the *Spodoptera* genus^1–3^. Despite their discovery more than 20 years ago, the details on the mode of action of Vip3A proteins as insecticidal toxins are not fully understood. Nevertheless, there is wide consensus on a general mechanism of Vip3 proteins consisting of a proteolytic activation in the insect lumen gut, binding to specific receptors on the midgut epithelium, and formation of ion channels which lead to the insect death^4,5^. A different, though not mutually exclusive mechanism, including an apoptotic pathway has also been suggested^3,4,6^. It has been recently proposed that Vip3A proteins act via mitochondrial swelling and caspase activation on Sf9 cells^7^. Although it has been shown that specific binding of Vip3A proteins can occur in non-susceptible species^4,8,9^, both mechanisms described above have in common that Vip3 and Cry proteins bind to different specific receptors and, therefore, their combination in the same pest management strategy is a promising tool to hinder the evolution of resistance to Cry toxins in addition to increasing the insecticidal potency and diversifying the range of target pests. Nowadays, the co-expression of Vip3 proteins with other entomopathogenic proteins, such as Cry toxins, are available to growers both in some Bt-based biopesticides and in pyramided Bt crops^10–16^, whereas promising applications based on nanoparticles and microencapsulation in bacterial cells are under exploration to render on-demand and more flexible green products based on Bt toxins^17–19^.

To date, 109 alleles of Vip3 proteins are known^13,20,21^, most of which have been tested and are active to a wide range of lepidopteran species with high selectivity at the species level. Whereas the spatial conformation in three domains of some of the Cry proteins is well established, Vip3 proteins do not share homology with any other known protein and the elucidation of their protein structure is a milestone yet to be achieved^22–25^. However, recent studies have shown that Vip3A are globular proteins with a quaternary structure^26–28^.

Alanine scanning is a successful technique for mapping crucial positions or epitopes in a protein and allows a greater insight into protein structure-function relationships. It consists of a systematic change, one by one, of the native amino acid residues to alanine residues. Alanine is the amino acid with the second smallest side chain, after glycine. It is composed of a chemically inert methyl group. The substitution of a residue to an alanine ‘removes’ the specific properties of a particular side chain while maintaining the β-carbon, ensuring the most frequent dihedral angle that connect the side chain with the amino acid backbone. Thus, the alanine mutagenesis simplifies the analysis of systematic residue substitutions in a protein sequence without changing the preferred secondary structure by introducing new chemical or steric properties that might blur the interpretation of the results^29,30^.

In the present work, an extensive examination of the Vip3Af1 primary structure was performed for the first time by means of the alanine scanning technique. A total of 558 out of the 788 residues of the Vip3Af1 sequence were changed to alanine and studied in detail. This is a first step in a better understanding of the Vip3A protein structure and the relationship to its biochemical hallmarks insecticidal activity.

## Materials and Methods

### Alanine Mutants Library

The *vip3Af1* gene (GenBank accession No. AJ872070.1) encoding the 788 amino acid protein Vip3Af1 (NCBI accession No. CAI43275, from now on: Vip3Af1(WT)), was modified to encode a His-tag sequence at the N-terminus. The alanine mutants library consisted of a total of 558 clones in *Escherichia coli* strain DH5α. Each clone differed from the others by a single amino acid codon which had been changed to an alanine codon from the Vip3Af1(WT) sequence cloned in the pMaab10 plasmid^31^. The changes were performed using the QuickChange Lightning Site-directed Mutagenesis Kit (Agilent Technologies, Inc.). The correct replacement of individual amino acid residues to the Ala residue was confirmed by sequencing of each individual mutant *vip3Af* gene. The sequence positions that were not changed to Ala were (Table 1): (i) the Met1 corresponding to the start codon; (ii) the N-terminus region (from amino acids 15 to 165) because, at the start of this project, this region was thought not to be involved in the insecticidal activity of the toxin; (iii) the 30 Ala residue positions in the Vip3Af1(WT), and (iv) 48 positions for which mutagenesis failed and no mutant protein could be obtained.

**Table 1:** List of missing positions of the Vip3Af1(WT) protein which could not be tested. Amino acid positions correspond to the Vip3Af1 protein sequence (NCBI accession No. CAI43275).

Description	Residues position	Num. of redidues
List of clones within the library with no expression of the modified-Vip3Af1 protein	Asn^5^, Ile^176^, Asn^180^, Phe^183^, Phe^286^, Arn^293^, Asp^302^, Tyr^409^, Phe^416^, Tyr^420^, Glu^458^, Leu^511^, Leu^520^, Asn^541^, Thr^564^, Gly^569^, Gly^639^, Ile^780^, Ser^786^	19
List of missing clones from the library:		
First amino acid (start codon)	Met^1^	1
Gap at the N-terminus region	From Ser^15^ to Thr^165^	151
Alanine in the wt sequence (Ala^x^)	x = 10, 12, 172, 189, 203, 216, 248, 252, 257, 280, 283, 285, 299, 336, 345, 351, 356, 375, 440, 462, 469, 496, 517, 554, 559, 572, 657, 670, 690, 725	30
Failed mutagenesis	Lys^7^, Val^179^, Lys^294^, Leu^296^, His^310^, Leu^311^, Asn^312^, Lys^313^, Glu^316^, Tyr^335^, Gly^340^, Glu^374^, Asp^386^, Leu^399^, Pro^417^, Glu^437^, Val^480^, Thr^518^, Asp^519^, Phe^581^, Tyr^595^, Ile^607^, Leu^609^, Lys^627^, Asp^628^, Thr^640^, Leu^649^, Asn^700^, Thr^706^, Arg^708^, Gln^709^, Ser^715^, Tyr^716^, Ser^720^, Ile^721^, Phe^729^, Arg^734^, Val^739^, Ser^748^, Ser^749^, Ser^751^, Phe^757^, Asn^762^, Asn^763^, Val^768^, Ser^711^, Phe^782^, Glu^783^	48
Total (missing positions)		230

### Protein Expression

For the initial screening, Vip3Af Ala-mutant clones and the Vip3Af1(WT) clone were expressed by picking up one single colony and inoculating it into a 3 ml of LB medium with ampicillin (100 µl/ml). After overnight incubation at 37 °C with mild shaking, 2 ml of this preculture was transferred to 20 ml of the same growth medium and incubated at 37 °C with mild shaking until the culture reached an OD_600_ of approximately 4.7. Aliquots of 100 and 1000 µl were taken and centrifuged for 20 min at 16,900 *g* at 4 °C. The supernatant was discarded and the pellets were stored at −20 °C until used, without any further treatment, for the initial toxicity assays.

For the rest of the work, since the *E. coli* strain DH5α is not inducible, selected clones were subjected to plasmid purification and the plasmid was transformed into the IPTG inducible strain *E. coli* WK6. All clones were grown and expressed as previously described^32^. After centrifugation, the crude extract supernatant was filtered through a 0.2 µm membrane before purification.

### Western Blot

After separation by SDS-PAGE, proteins were electroblotted in duplicate onto nitrocellulose membranes. Membranes were blocked with 3% of bovine serum albumin (BSA) in PBST buffer (Phosphate Buffered Saline with 0.1% Tween 20), and after washing three times with PBST, membranes were incubated either with a rabbit polyclonal antibody raised against anti-Vip3Aa (full length, purified by affinity chromatography using HisTag columns), which cross-reacted against Vip3Af proteins (1:2000 dilution), or with a monoclonal anti-His antibody (1:5000 dilution). Membranes incubated with the anti-Vip3 antibody were probed with an anti-rabbit IgG-conjugated horseradish peroxidase (1:5000 dilution) and bands were visualized with a chemiluminescence detection kit (RPN2209; GE Healthcare) using an ImageQuant LAS400 image analyser (GE Healthcare). Membranes incubated with the anti-His antibody were probed with an anti-mice IgG conjugated to alkaline phosphatase (1:2000 dilution) and the results were visualized with the NBT/BCIP color detection reagent.

### Protein Purification

Purification of the crude extracts was carried out with a metal-chelate affinity chromatography as previously described by Hernández-Martínez *et al*.^33^ using HisTrap FF columns (GE Healthcare). Fractions of 1 ml were eluted from the column and collected in tubes containing 50 µl of 0.1 M EDTA. The fractions containing Vip3Af were pooled and dialyzed against 20 mM Tris, 150 mM NaCl, 5 mM EDTA, pH 8.6, before storage at −20 °C.

For purification by isoelectric point precipitation, the pH of the clarified crude extracts was lowered to 4.7 with 0.1 M acetic acid while stirring on ice. The precipitated protein was pelleted by centrifugation and stored at −20 °C.

### Insect Rearing and Bioassays

Insect colonies of *Spodoptera frugiperda* and *Agrotis segetum* (Lepidoptera: Noctuidae) were reared on a semi-synthetic diet^34^ in a rearing chamber under controlled conditions of temperature, humidity and photoperiod (25 ± 2 °C, 70 ± 5% RH and 16:8 h L:D).

Surface contamination bioassays were conducted by applying 50 µl of the Vip3Af protein sample on a 2 cm^2^ diameter well in multi-well plates filled with the semi-synthetic diet and let dry. A single neonate larvae was used in each well. Bioassay plates were maintained in the insect rearing chamber. All bioassays were scored at 7 days for mortality and functional mortality (dead larvae plus stunt larvae at L1). Three types of bioassays were conducted:

(i) For the screening of the alanine mutants library, pellets from 100 µl of bacterial cultures were resuspended in 1 ml of PBS. Every clone was bioassayed at a single concentration (corresponding approximatively to 150–190 ng/cm^2^ of Vip3Af protein) using 16 individualised neonates of *S. frugiperda*. A negative control (*E. coli* DH5α without the pMAAB plasmid containing the *vip3Af* gene) and a positive control (*E. coli* expressing the Vip3Af1(WT) protein) were always done in parallel. Mutants that displayed no insecticidal activity were selected for having a mutation considered critical for the protein function. Selected mutants were screened twice. In order to dismiss a false positive result, the expression of Vip3Af protein on these samples was checked by SDS-PAGE.

(ii) To obtain LC_50_ values of the Vip3Af1(WT) to be used as a reference for the semi-quantitative bioassays, quantitative toxicity assays were performed against *S. frugiperda* and *A. segetum* with Vip3Af1(WT) partially purified by isoelectric point precipitation, since it was previously reported that affinity purification with Ni-columns can affect the insecticidal activity of Vip3A proteins^33^. The precipitated Vip3Af1(WT) protein was solubilised in 20 mM Tris, 300 mM NaCl, pH 9, and then serially diluted to 7 different concentrations. The solubilisation buffer was used as a control. Regression curves were estimated from at least three replicates using the POLO-PC probit analysis program^35^.

(iii) For the semi-quantitative bioassays, Vip3Af mutant proteins were expressed and partially purified by isoelectric point precipitation. As in the quantitative bioassays above, the precipitated Vip3Af proteins were solubilised in 20 mM Tris, 300 mM NaCl, pH 9, and tested at a concentration of 1 µg/cm^2^, which is 130-fold higher than the LC_50_ for *S. frugiperda* and 28-fold higher than the LC_50_ for *A. segetum* (Table 2). A total of 16 neonates of *S. frugiperda* were assayed for each mutant. The Vip3Af1(WT) protein purified by isoelectric point precipitation served as a positive control. The mutant proteins for which loss of insecticidal activity was confirmed on *S. frugiperda*, were also bioassayed against *A. segetum* in the same way. Bioassays were repeated two to three times.

**Table 2:** Quantitative parameters from concentration-mortality responses (at 7 days) of Vip3Af1(WT) partially purified by isoelectric point precipitation on *S. frugiperda* and *A. segetum*.

Lepidopteran species	Regression line		LC_50_ (ng/cm^2^)	95% FL^†^		Goodness of fit value χ^2^
	Slope ± SE	A^*^ ± SE		Lower	Upper	
*S. frugiperda*	1.0 ± 0.1	4.2 ± 0.1	7.6	4.9	11.0	4.3
*A. segetum*	0.9 ± 0.1	3.5 ± 0.3	35.2	19.1	58.9	4.8

^†^FL: Fiducial limits.

^*^a: intercept.

### Midgut Juice Preparation

*Spodoptera frugiperda* and *A. segetum* 5^th^ instar larvae were dissected on ice and the bolus content, along with peritrophic membrane enclosing it, was collected. Once the peritrophic membrane was removed, the bolus contents from eight to ten larvae were mixed and centrifuged at 4 °C for 10 min at 16,000 *g*. The supernatant was collected and distributed in small aliquots, immediately frozen in liquid nitrogen and stored at −80 °C. Total protein concentration in the midgut juice was quantified with Bradford reagent using BSA as standard^36^.

### Proteolytic Pattern Assays

Affinity-purified Vip3Af proteins (5 µg) were incubated with 5% trypsin or with midgut juice from either *S. frugiperda* or *A. segetum* at 0.4% in a final volume of 40 µl. After 1 h incubation at 37 °C, PMSF was added at a final concentration of 250 µM, followed by SDS-PAGE loading buffer (0.2 M Tris-HCl pH 6.8, 1 M sucrose, 5 mM EDTA, 0.1% bromophenol blue, 2.5% SDS, and 5% β-mercaptoethanol) (2:1, sample:loading buffer) and then the samples were heated at 99 °C for 5 min. Samples were either run immediately in 12% SDS-PAGE or frozen in liquid nitrogen and stored at −20 °C for further analysis. The Vip3Af1(WT) was included as an internal control in all reactions. Reactions without the addition of trypsin or midgut juice were always conducted in parallel as a control for the potential thermolability of the mutant proteins. The whole assay was replicated twice. The size of the SDS-PAGE main bands in each protein sample was determined using the TotalLab 1D v 13.01 software.

To test whether the proteolysis patterns could be affected by the presence of SDS in the loading buffer^37^, mutants W552A (pattern “b”) and E483A (pattern “f”) were subjected to incubation with 20% trypsin for 1 min to up to 3 days at 30 °C. The samples were processed and analysed by SDS-PAGE as indicated above. Additionally, the reaction mixtures after 30 min incubation with 5% trypsin at 37 °C were subjected to gel filtration chromatography in a Superdex-200 10/300 GL column using an ÄKTA explorer 100 chromatography system (GE Healthcare Life Sciences, Uppsala, Sweden) equilibrated and eluted with 20 mM Tris-HCl, 150 mM NaCl, pH 9, at a flow rate of 0.75 ml/min.

### Peptide Identification

Trypsin-generated fragments from the affinity-purified Vip3Af1(WT) were electrophoretically separated in 12% SDS-PAGE. For Edman degradation analysis, proteins in the gel were transferred onto a PVDF membrane. Protein bands were then cut out and sent for Edman degradation. N-terminal amino acid sequencing was performed by using a Procise 494 (Applied Biosystems) at CIB-CSIC (Madrid, Spain).

For the peptide mass fingerprinting, protein bands were directly cut out from the gel and digested with trypsin. The peptide mass and sequence was determined by liquid chromatography and tandem mass spectrometry (LC-MS/MS) in a nanoESI qQTOF (5600 TripleTOF, ABSCIEX) at the proteomics facility of the SCSIE (Servei Central de Suport a la Investigació Experimental), at the University of Valencia (Valencia, Spain). The mass transitions were scanned first from 350–1250 m/z and then followed by a second scan from 100–1500 m/z. The peptides sequence identified were compared to the Vip3Af1(WT) protein sequence to match the region corresponding to each SDS-PAGE proteolytic band. Expected molecular weights were calculated using the online SIB Compute pI/Mw tool^38^.

### Intrinsic Fluorescence Emission Spectra

The intrinsic fluorescence of the Vip3Af proteins, before and after trypsin treatment, was checked in a Varian Cary Eclipse fluorimeter (Agilent Technologies, Australia) with an excitation at 280 nm (excitation slit of 5 nm) and recording the emission spectra from 300 to 450 nm (emission slit of 20 nm). Fluorescence of the affinity-purified proteins (5–10 µg) in 20 mM Tris, 300 mM NaCl, pH 9, was measured in a quartz cuvette in a final volume of 1.3 ml. After recording the spectra, the samples were subjected to trypsin treatment (20%, at 37 °C for 1 h). Graphic curves represent the average of three scans. The curves were smoothed with the moving average algorithm.

### In silico prediction of the 3D structure of Vip3Af1(WT)

The *ab initio* modelling of the full sequence of the Vip3Af1(WT) protein was done using the fully automated server Robetta available online using guinzo domain prediction (http://robetta.bakerlab.org). The prediction of disulfide bonds were done using Disulfind online server (http://disulfind.dsi.unifi.it)^39^ and the DiANNA 1.1 web server online tool (http://clavius.bc.edu/~clotelab/DiANNA/)^40^.

### Data availability

The raw data used to support the findings of this study and the supplementary information are available from the corresponding author upon reasonable request.

## Results

### Screening of the Alanine Mutants Collection

**Figure 1:**
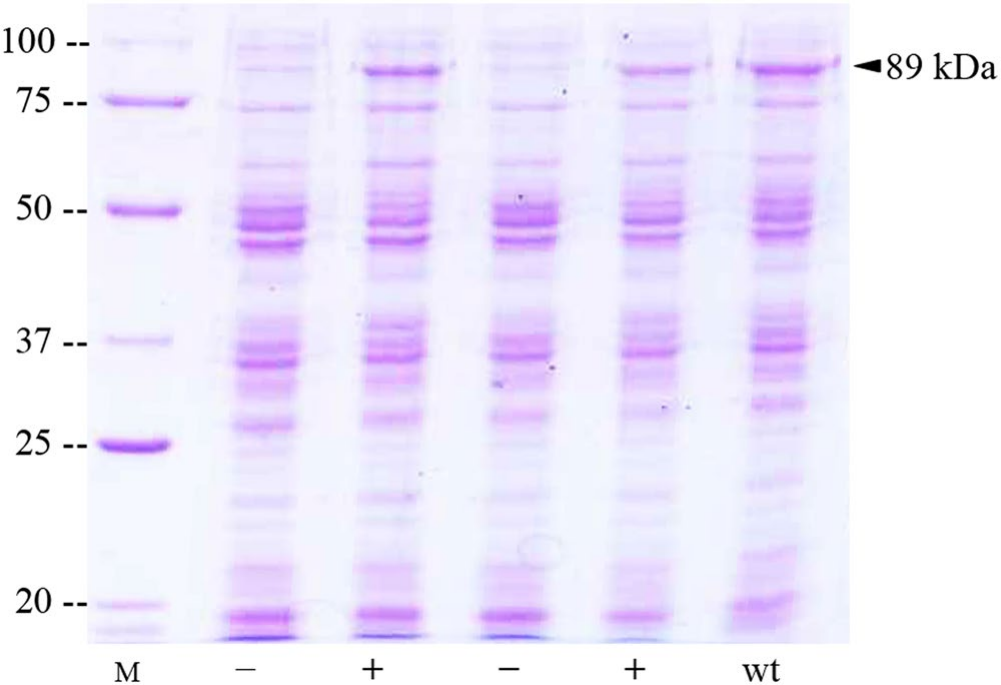
Detection of the expression of Vip3Af proteins (89 kDa) in the collection of Ala-mutants used in the screening. Direct broth (10 µl of the culture adjusted to an OD600 of 4.7) was loaded and subjected to SDS-PAGE. “M”: molecular weight marker (kDa); minus sign (−): absence of Vip3Af expression; plus sign (+): Vip3Af expression, and “wt”: Vip3Af1(WT) as a positive control.

**Figure 2:**
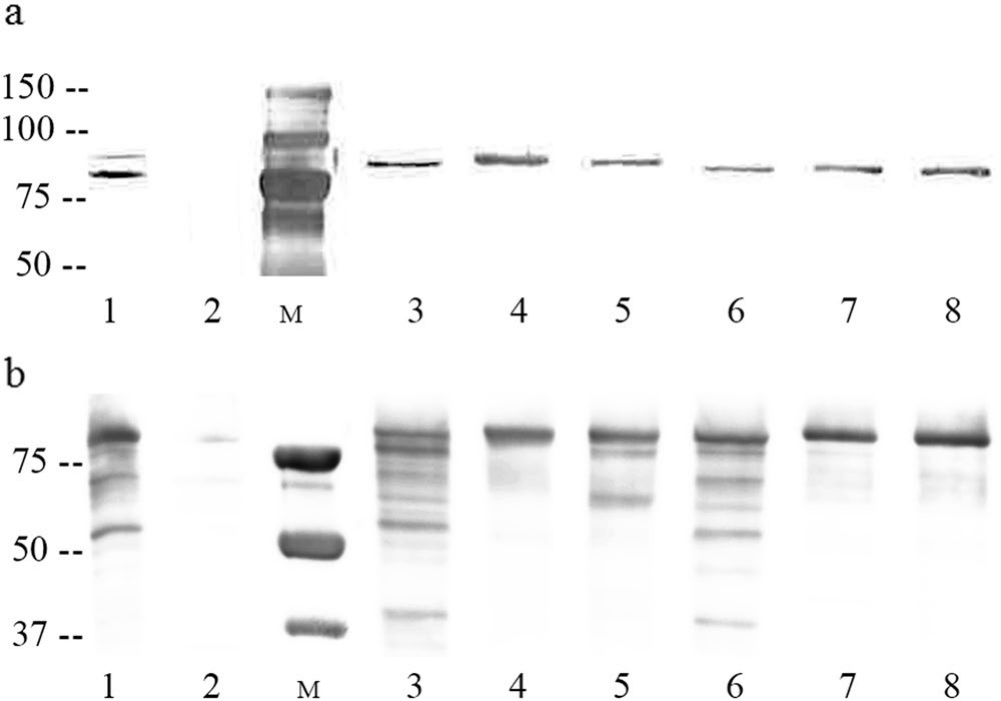
Detection of Vip3Af in the crude extract after isoelectric point precipitation. Membranes were probed with monocolonal antibodies against histidine for His-tag detection (**a**) and with polyclonal antibodies against Vip3A proteins (**b**). Lane 1: Vip3Af1(WT) as a positive control; lane 2: *E. coli* wk6Ø as a negative control; lanes 3 to 8: mutants Y272A, W552A, Y719A, M238A, G689A, and E483A, respectively. “M”: molecular weight marker (kDa). Full-length blots and Comassie blue stained gels are presented in Supplementary Figure 1.

As a first step to determine the positions critical for the insecticidal activity of the Vip3Af1(WT), a quick screening was carried out on *S. frugiperda* neonates with *E. coli* cells expressing each of the 588 clones (Table 1). A total of 54 clones were found to show a substantial decrease of the insecticidal activity. In the experimental conditions in which the wild type Vip3Af1 protein (Vip3Af (WT)) produced a mortality higher than 80% and a functional mortality of 100%, these 54 clones produced a mortality lower than 25% and a functional mortality lower than 45%. The expected mutations in these clones were confirmed by PCR amplification and sequencing (Supplementary Table S1). After verifying Vip3Af expression in those clones with low activity by SDS-PAGE (Fig. 1) and Western blot (Fig. 2) (full length images can be found in Supplementary Fig. S1), 19 were found not to express the protein (Table 1). Since sequencing of the *vip3Af* gene and vector indicated no sequence error, we infer that these positions might be critical for the stability of the wild type protein or its expression.

**Figure 3:**
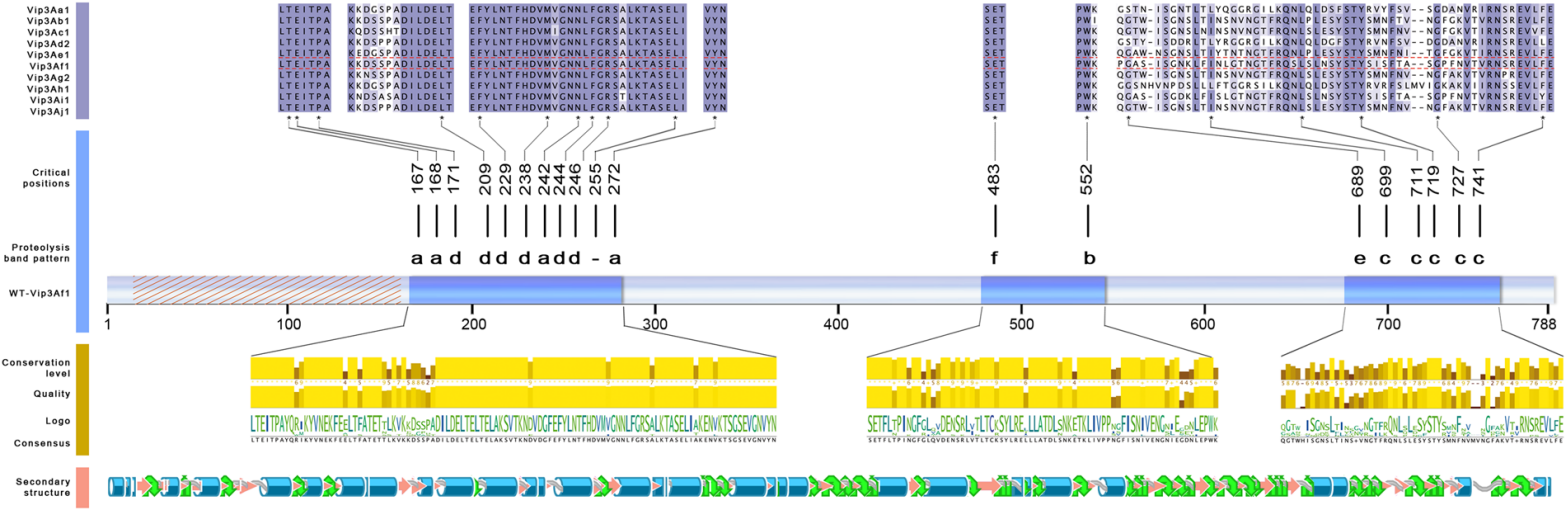
Critical amino acid positions for the insecticidal activity of Vip3Af1. The Vip3Af1(WT) sequence is represented by the pale blue bar. The dashed region was not subjected to alanine scanning; dark blue segments in the protein bar are the regions where these critical positions are clustered. Above the protein bar, letters “a” to “f” illustrate the different proteolysis profiles displayed by the proteins with the substitutions to Ala in these positions (see Fig. 4). Conservation of these sites (upper part of the figure) was evaluated by Clustal Omega msa^68^ of different Vip3A proteins from *Bacillus thuringiensis*: Vip3Aa1 (GenBank accession number AAC37036), Vip3Ab1 (accession number AAR40284), Vip3Ac1 (named PS49C with Seq. ID 7 in U.S. patent application 20.040.128.716 (Narva and Merlo)), Vip3Ad2 (accession number CAI43276), Vip3Ae1 (accession number CAI43277), Vip3Af1 (accession number CAI43275), Vip3Ag2 (accession number ACL97352), Vip3Ah1 (accession number ABH10614), Vip3Ai1 (accession number KC156693) and Vip3Aj1 (accession number KF826717). The msa is coloured according to BLOSUM62 colour scheme. Amino acid conservation, quality (BLOSUM62 score based on observed substitutions), logo visualisation (Buried index: dark green (buried), light green (exposed) and consensus sequence as visualised by Jalview^69^ is shown below the pale blue bar. The lower panel represents the predicted secondary structure using Geneious v. 6.0^70^: blue cylinders are α-helixes, pink arrows are β-sheets, green arrows are turns, and grey waves represents coils.

To confirm the above data, a more accurate semi-quantitative bioassay with partially purified Vip3Af proteins was carried out with the selected 35 clones (those which decreased the insecticidal activity and for which the expression of the Vip3Af protein was confirmed). In a first step, all 35 clones were tested against *S. frugiperda*. Then, the clones exhibiting the most drastic decrease in toxicity against this insect species were subsequently tested against *A. segetum* (Table 3). Figure 3 shows the graphical representation of the distribution of the most drastic positions (the 19 clones causing mortality lower than 50% to *S. frugiperda* in Table 3). Except for two of these positions (residues 483 and 552), the remaining positions fell into two clusters, one between residues 167 to 272, and the other between residues 689 and 741.

**Table 3:** Insecticidal activity of the Vip3Af1(WT) and the mutant proteins on *S. frugiperda* and *A. segetum* at a concentration of 1 µg/cm^2^ (average of two replicates) with indication of the proteolytic band pattern.

Vip3Af protein	Proteolysis band pattern	*S. frugiperda*		*A. segetum*	
		% M	% fM	% M	% fM
Vip3Af1(WT)	a	72	100	60	100
T167A	a	3	6	31	94
E168A	a	16	22	6	6
T170A	a	56	94		
P171A	d	34	72		
E184A	a	59	100		
L194A	ND	81	100		
L209A	d	47	94		
L212A	a	81	100		
L215A	d	72	97		
F229A	d	22	56		
Y230A	d	59	100		
M238A	d	28	59	13	35
N242A	a	44	91	59	100
F244A	d	49	90	72	94
R246A	d	41	97	47	100
K250A	a	91	100		
E254A	a	62	100		
L255A	ND	41	81		
V261A	a	59	97		
N270A	a	78	100		
Y272A	a	10	19	0	0
V277A	ND	67	100		
L287A	a	66	100		
I301A	a	58	73		
M307A	a	63	100		
E483A	f	21	61	63	100
F485A	a	59	100		
W552A	b	13	13	13	16
G689A	e	41	69	16	26
I699A	c	29	33	0	0
L711A	c	44	100	44	72
Y719A	c	22	25	3	6
G727A	c	27	27	0	0
F741A	c	28	94	38	56
H779A	ND	56	59		

Bioassays were scored after 7 days. M%: mortality. fM%: functional mortality (dead larvae plus stunt larvae at L1). ND: not determined due to the lack of His-tag in the protein.

### Proteolytic cleavage of the wild type and mutant Vip3Af proteins

**Figure 4:**
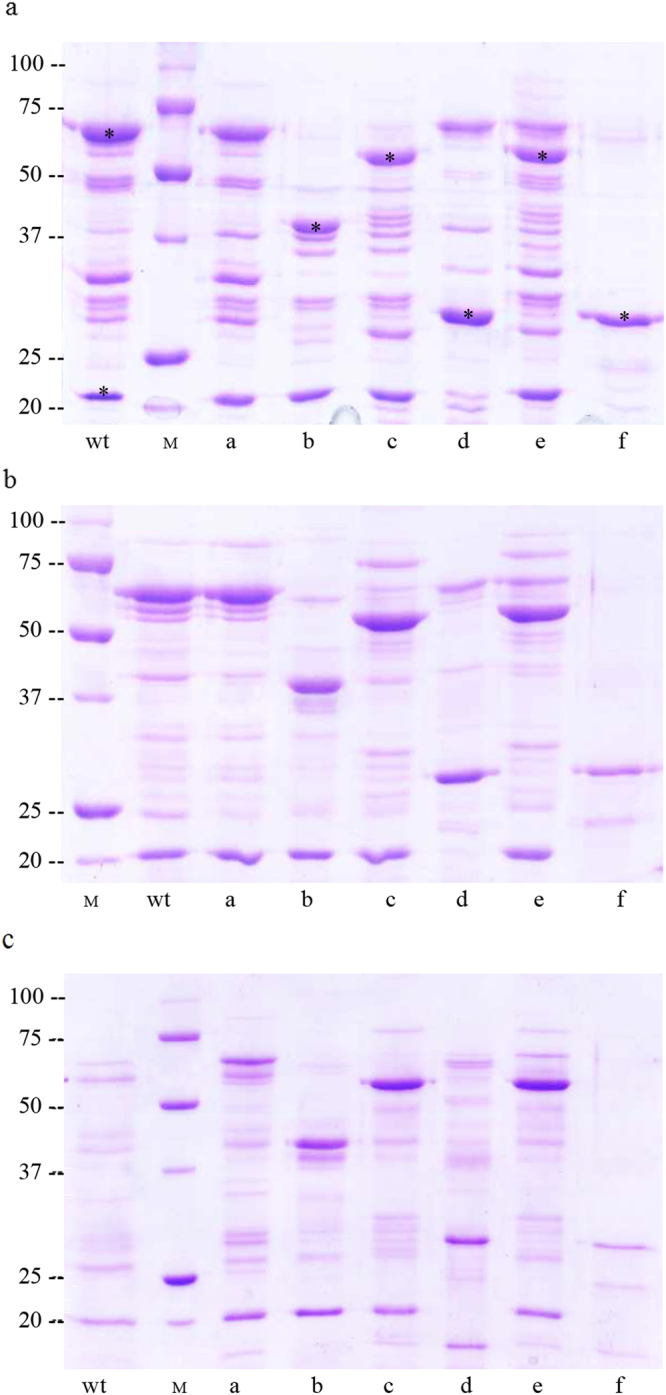
Representative proteolytic band patterns of Vip3Af1(WT) and selected mutant proteins after SDS-PAGE. Protein samples (5 μg) were incubated with 5% trypsin (wt/wt) (**a**), 0.4% midgut juice from *S. frugiperda* (**b**) or 0.4% midgut juice from *A. segetum* (**c**) (wt/wt, midgut juice total protein/Vip). Incubations were performed at 37 °C for 1 h. Lanes “wt”: Vip3Af1(WT), “M”: molecular weight marker (kDa), “a to “f”: mutants Y272A, W552A, Y719A, M238A, G689A and E483A, respectively. The different proteolysis profiles are defined according to their main protein bands after SDS-PAGE as follows: Pattern “a” (corresponding to the “wt): 62 kDa and 20 kDa; pattern “b”: 40 kDa and 20 kDa; pattern “c”: 53 kDa and 20 kDa; pattern “d”: 62 kDa and 27 kDa; pattern “e”: 62 kDa, 57 kDa and 20 kDa, and pattern “f”: 27 kDa. “*” indicates the bands analysed for peptide identification either by EDMAN degradation or by peptide mass fingerprinting.

To indirectly assess whether the change to Ala could affect the structure of the protoxin, the 35 mutant proteins selected in the screening for decreased insecticidal activity were subjected to proteolytic treatment and classified according to their proteolysis band pattern. Six different band patterns were revealed by SDS PAGE (Fig. 4). The patterns were comparable no matter whether they were obtained with bovine trypsin or with midgut juice from either *S. frugiperda* or *A. segetum*.

Different sizes of the most conspicuous bands characterise these patterns, especially the bands corresponding to the 62 kDa and 20 kDa fragments, the two more intense bands in the Vip3Af1(WT), which characterize the pattern “a” and which are generated by the action of proteases on the primary cleavage site^37^. The 62 kDa band was not among the main bands in patterns “b”, “c”, “e” and “f”, and the 20 kDa band was not present in patterns “d” and “f”, which showed a major band of 27 kDa. Patterns “b”, “e” and “f” were only represented once among the 35 pre-selected mutants. It is also worth to note that patterns “a” and “d” (both maintaining the 62 kDa band) were found in those mutants clustering in the first half of the protein, whereas patterns “c” and “e” (both being very similar except for the faint presence of the 62 kDa band in “e”) were found in mutants clustering near the C-terminus. Patterns “b” and “f” were found in the two mutants not included in these two clusters.

**Figure 5:**
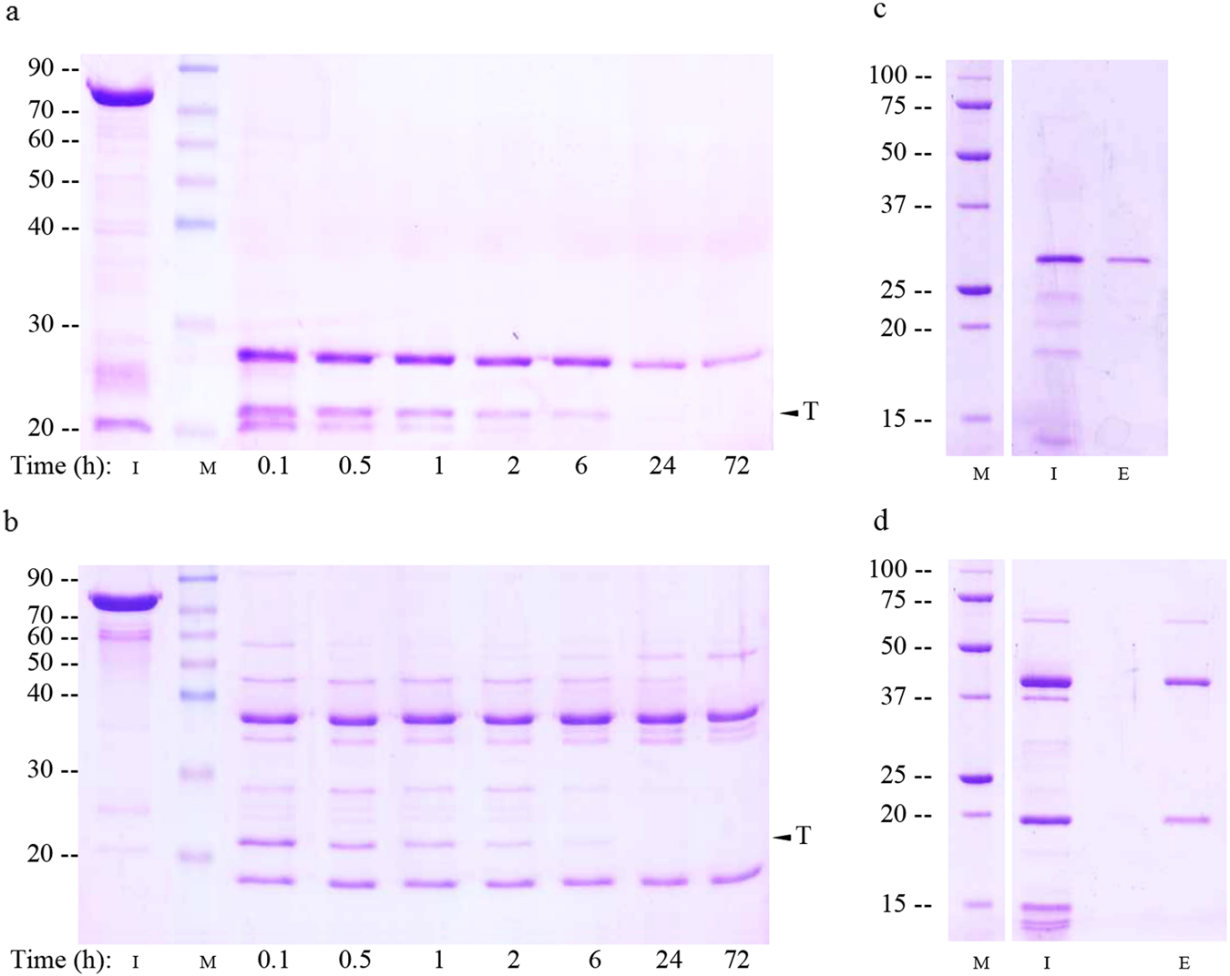
Analysis of the effect of the SDS on the proteolytic pattern of Vip3Af-mutant proteins E483A (pattern “f”, panels a and c) and W552A (“pattern b”, panels b and d) after trypsin treatment. Protein samples were treated with 20% trypsin at 30 °C for the kinetics analysis (panels a and b); each lane corresponds to 3 µg of protein sample. For SDS-PAGE analysis of the size exclusion chromatography elution peak (panels c and d), protein samples were treated with 5% trypsin for 30 min at 37 °C, injected into a Superdex-200 10/300 GL column and the elution fractions subjected to SDS-PAGE. Panels c and d show the input sample and the elution fraction containing the protein peak as revealed by SDS-PAGE. “M”: molecular weight marker (kDa), “I”: Protoxin in a and b, sample input in c and d, “E”: Elution peak. “T”: Trypsin.

It has been recently shown that proteases, in the presence of SDS, can act on secondary cleavage sites of Vip3Aa in the interval between the moment that the loading buffer is added and the moment that the proteins are heat denatured^37^. To test whether our proteolysis patterns could be affected by the presence of SDS in the loading buffer, mutants W552A (pattern “b”) and E483A (pattern “f”) were subjected to incubation with trypsin for up to 3 days (these two mutants were chosen because they presented the proteolytic patterns most different to that of the wild type protein, which may make one suspect that they were the result of SDS denaturation while terminating the reaction to prepare it for SDS-PAGE). The results showed that, regarding the major bands, the pattern that appeared after 1 min incubation was the same as the one obtained after 3-days incubation (when no trypsin remains in the reaction mixture) (Fig. 5a,b). Similarly, the major bands pattern obtained for these two mutants did not change after subjecting the reaction mixture to gel filtration chromatography, indicating that removal of trypsin before SDS-PAGE does not have an effect on the major bands that characterize the proteolysis pattern (Fig. 5c,d). Minor bands are probably a consequence of the SDS effect on the protein. Therefore, under the experimental conditions used, these band patterns reflect the digestion of these mutated protoxins to proteases *in vitro*.

### N-terminal sequence analysis of tryptic fragments and peptide identification

Some proteolytic fragments were subjected to N-terminal sequencing to determine their position in the protein (see Fig. 4). The 20 kDa fragment, obtained after trypsin treatment of the Vip3Af1(WT) (and present in patterns “a”, “d”, and “e”), yielded the sequence ALPSF, the first amino acid corresponding to Ala^12^. The N-terminal sequence of the 62 kDa was DXXPA, which only matched with the query sequence on DSSPA (D = Asp^199^). The protein is thus cleaved after Lys^198^, within a highly conserved region rich in lysine residues, cleaving the protein in two main fragments of 20 kDa and 62 kDa. The N-terminal sequence of the 27 kDa fragment obtained after trypsin treatment of the E483A mutant (pattern f) was IVPPS, which perfectly matches with the sequence starting with Ile^528^ and, considering the molecular weight of the fragment, it spans from Ile^528^ till almost the C-terminus of the protein. This result was in agreement with the region identified using the peptide mass fingerprinting. The peptide identification of the other bands highlighted with an asterisk in Fig. 4 shows that the 40 kDa band from the mutant W552A (pattern b) is derived from the central region of the protein, matching small peptides from Ser^247^ to Lys^602^, a region that corresponds to a theoretical size of 39.8 kDa. Similarly, the 55 kDa band present in “pattern c” matched different small peptides spanning the region from Tyr^178^ to Lys^661^. The peptide identification of the 27 kDa band from the mutant M238A (pattern d) and the 55 kDa band from mutant G689A (the only representative of “pattern e”) did not give clear results. However, considering that the proteolytic patterns are the result of changes in the conformation of the molecule that expose hidden cleavage sites, it is not too risky to assume that they correspond to the homologous bands from “pattern f” and “pattern c”, respectively.

### Emission Spectra from Vip3Af1(WT) and Vip3Af-selected mutants

**Figure 6:**
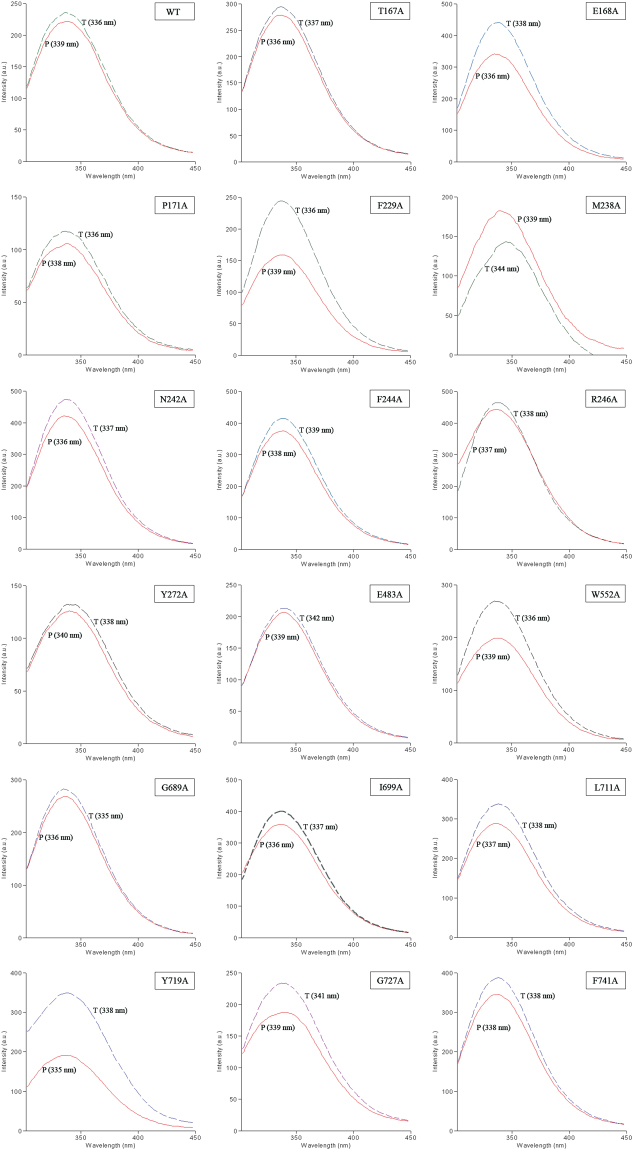
Emission spectra of the intrinsic fluorescence of Vip3Af1(WT) and mutant Vip3Af proteins with reduction of their insecticidal activity. Proteins (3–8 µg/mL) were excited at 280 nm and the emission was scanned from 300 to 450 nm. “P”: protoxin; “T”: protoxins treated with 20% trypsin (wt/wt) at 37 °C for 1 h. The maximum emission peak is indicated within brackets.

An indirect measure of the spatial conformation and folding of the Vip3Af1(WT) and the selected mutant proteins was obtained by analysing the intrinsic fluorescence emission spectra of the protoxin and the toxin forms (Fig. 6). All Vip3Af protoxins and the processed forms showed emission maxima below 348 nm (the maximum expected for free Trp in water^41^), indicating that most Trp are buried into the hydrophobic core of the protein. The existing Trp residues in the Vip3Af are located in the C-terminal end at positions W552, W658 and W684. The fluorescence emission spectra of the protoxin forms were all essentially similar to that of the wild type, suggesting that the substitution had a minimal effect on the conformation of the protein. In the transition from protoxin to toxin, there is only one case (M238A) for which the quantum yield decreases, along with the largest shift to the red (to 344 nm, approaching the expected emission maximum for the free Trp in aqueous solution), suggesting a change in the surrounding of the Trp residues to higher polarity, perhaps by being almost completely exposed to the aqueous buffer. Two other substitutions (E483A and G272A) also provoked a considerable shift to the red compared with the trypsin-treated wild type protein. Except for M238A, the transition from protoxin to toxin renders either practically no increase in the quantum yield (as in the case of the wild type protein) or a significant increase (as in the case of F229A). The latter situation suggests changes to a less polar environment.

### Structure prediction of the Vip3Af1(WT)

The *in silico* modelling of the Vip3Af1(WT) resulted in a structure parsed into 5 different domains, three of which are modelled by homology whereas the other two are modelled *de novo*. Domain 1 spans up to amino acid 188 (*de novo*, msa confidence level 0.18), Domain 2 spans from position 189 to 272 (homology, confidence level 0.20), Domain 3 extends from amino acid 273 to 542 (*de novo*, msa domain confidence level 1.01), Domain 4 is predicted from residue 543 to residue 715 (homology, confidence level 0.42) and Domain 5 spans from amino acid 716 till the end of the protein (homology, confidence level 0.83).

**Figure 7:**
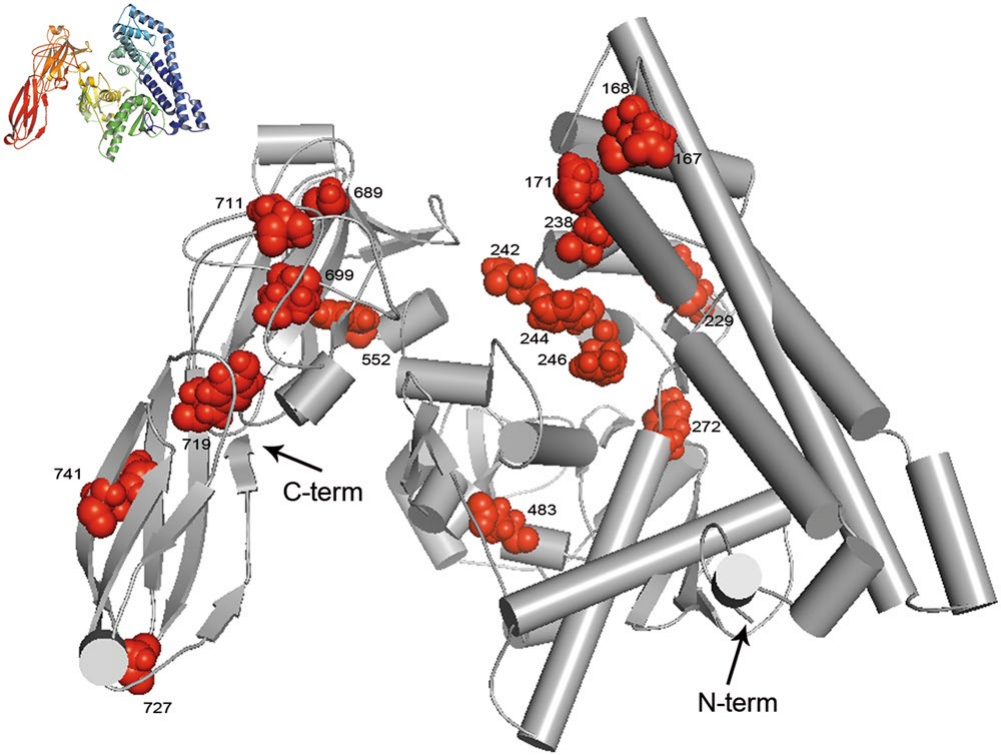
Representation of the critical positions in a 3D conformation of the Vip3Af1(WT) modelled *ab initio* using Robetta (confidence level of the domains conforming the model vary from 0.18 to 0.83) visualised with PyMOL v 1.8^71^. The structure in the upper left corner is coloured from blue (N-term) to red (C-term) and serves as a guide to visualize the 5 predicted domains.

The three-dimensional structure of this model (Fig. 7 and Supplementary 3D File online)) identifies two main regions: The N-terminus depicted mostly by α-helix structures, and the C-terminus with greater prevalence of ß-sheet structures. The central region of the protein is mainly formed by disordered structures. The prediction of whether residues, represented in the alignment shown in Fig. 3, were exposed or buried was conducted using the Conseq server^42^. No disulphide bonds are expected in any of the 3 cysteine residues along the Vip3Af1(WT) sequence according to the two online servers employed; nevertheless, DiANNA server predicted C401 as a half-cystine with a score of 0.53.

## Discussion

A total of 558 out of 788 residues of the Vip3Af1(WT) were analysed for their specific contribution to the insecticidal selectivity and potency, and to protein stability, by means of alanine scanning. Most of the substitutions constituted neutral mutations, stressing the high level of resilience and adaptability of Vip3A proteins to preserve protein function and homeostasis, even when most of these changes involved the substitution of highly conserved residues among the Vip3A subfamily. Only around 10% of the residues analysed play a crucial role in either protein stability, protein folding, proteolytic processing or insecticidal activity of the Vip3Af1(WT). In all, we have detected over 50 substitutions affecting the function/stability of the protein, with 19 of them compromising protein expression or, alternatively, rendering such unstable proteins that were degraded immediately after their expression, since it was not possible to detect the protein expression in SDS-PAGE (Fig. 1). The substitutions affecting the insecticidal activity against *S. frugiperda* did so to different degrees (Table 3). Further characterization of these variants by protease analysis was performed in order to indirectly assess the impact of the substituted residues on the tertiary structure of the protein. The clustering of the 19 most critical positions affecting insecticidal activity (mortality less than 50% in Table 3) revealed two main ‘*hot spots’* along the Vip3Af1(WT) sequence, one located near the N-terminus (Leu^167^–Tyr^272^) and the other near the C-terminus (Gly^689^–Phe^741^) (Fig. 3). These clusters correspond to the end of domain 1 and the whole domain 2, and the end of domain 4 and beginning of domain 5, predicted in the *ab initio* tertiary structure, respectively (Fig. 6). Likewise, the different proteolytic patterns clustered in preferred regions of the sequence: proteolytic patterns “a” and “d” were only present in the cluster in the N-terminal region, whereas pattern “c” was only represented in the C-terminus cluster along with pattern “e” (which indeed is similar to pattern “c” with an extra 62 kDa band). Most of the changes compromising the insecticidal activity consisted of a variety of hydrophobic amino acids. Alanine is the smallest of the hydrophobic residues, thus, a hydrophobic bulk decrease might imply local steric modifications negatively affecting the protein function.

It is very likely that mutants displaying profile “a” do not alter the tertiary structure of the protein, since this is the band pattern obtained for the wild type protein. Thus, the loss of more than 50% insecticidal activity in four of these pattern “a” mutants is likely due to the change in their respective residues without leading to a misfolded protein. This change might be itself critical for the insecticidal function or otherwise interfere with critical interactions with other molecules. Pattern “a” was characterised by the main bands of 62 kDa and 20 kDa, similarly to the 65 kDa and 22 kDa bands initially described for the Vip3Aa1 by Estruch and Yu^3^. Peptide identification confirmed previous reports^3,28^ in that the protein sequence is split in two after Lys^198^, giving rise to two main fragments which match the C-terminal and the N-terminal parts of the protein. There is evidence that, after proteolytic digestion, these two main fragments co-elute after gel permeation chromatography, indicating that they remain bound after cleavage^8,28,37^.

The other band patterns different from “a” (i.e. patterns “b” to “f”) reveal changes in the structure that make secondary cleavage sites (those sites different from the main cleavage site after Lys^198^) more readily available to proteases. The fact that the proteolytic patterns obtained are similar after trypsin treatment and after midgut juice treatment, from both *S. frugiperda* and *A. segetum* (Fig. 4), suggests that the decrease of toxicity of these mutants is related to the instability to proteases *in vivo*. Since the fluorescence emission spectra of the protoxin form essentially did not change in the mutants compared to the wild type, very likely the effect of the substitution on the conformation of the protein must be minimal. However, the difference between the emission maximum of the toxin form of the wild type and mutants M238A, E483A and G727A indicates more drastic changes in the conformation of the protein, making the Trp residues being more exposed to the solvent. The absence of a major folding change of the Vip3 proteins after activation is in agreement with the recently published study on the 3D topology of the Vip3Ag4 protein^27^. An exception to this general effect is mutant E483A, in which a drastic change in the protein fold after activation takes place as revealed by the unique proteolytic band of only 27 kDa (Fig. 5). This single fragment contains the three Trp residues of the Vip3Af, suggesting that the partial folding of the β-structure in the C-terminus remains unchanged.

The largest fragment from the trypsin treatment, the 62–66 kDa polypeptide, was formerly considered to be the core active toxin^1,3–5,8,12,33,37,43–49^. However, it has been recently shown that the 20 kDa fragment is required for toxicity, presumably by protecting the 62–66 kDa polypeptide from protease degradation^28^. Zack *et al*. have shown, using chimeric Vip3A/Vip3B proteins for which the N-terminal 20 kDa fragment had been exchanged, that the interaction of the 20 kDa fragment with the 66–66 kDa fragment from their original protein was essential for its correct function^28^. The chimeric Vip3A/Vip3B proteins had completely lost their insecticidal activity. Here, we have found that some of the mutants with proteolytic patterns lacking the 62 kDa band still retain some insecticidal activity, which could suggest that fragments smaller than 62 kDa are toxic (Table 3). However, since the toxicity of these mutants was tested with the protoxin form and not with the processed protein, we cannot attribute the toxicity of the mutants to the fragments smaller than 62 kDa. It is very likely that the toxicity observed *in vivo* with the protoxin is due to the transient formation of the 20 kDa/62 kDa complex (caused by cleavage at the primary site^37^) before the final processing to smaller fragments. This could be tested by directly feeding the insects with the mutant proteins after trypsin activation.

Regarding the smallest fragment of 19–22 kDa at the N-terminus, there is agreement in that it contains a signal peptide involving the first 34 residues, which is not removed after protein secretion^50–53^. However, Doss *et al*.^50^ suggested a putative cleavage site between residue Thr^10^ and Arg^11^ in Vip3Aa based on the S score prediction. Removal of N-terminal 12 amino acids (in Arg^12^) was shown in Vip3Ab1^28^ and we have here shown that the 20 kDa N-terminal fragment of Vip3Af1 is also cleaved after Arg^11^.

Few attempts have been made to elucidate the role of the N-terminus of the Vip3A proteins other than identifying the presence of a predicted signal peptide^43,52,54–56^ and, among these, the results are controversial: deletion of the first 200 residues in Vip3Aa led to opposite results, from complete suppression of insecticidal activity and total sensitivity to trypsin digestion, to an increase of up to 2.8-fold in the toxicity against different caterpillar species, both when tested as the purified toxin and when expressed in a tobacco transgenic line^1,12,43^.

The lack or decrease of toxicity in Vip3Aa mutants with C-terminal modifications was often related to an increase in the sensitivity towards proteases in the gut environment of susceptible insects. A triple mutation in the C-terminal sequence of Vip3Aa1, and also the Vip3Aa3 protein, which lacks the 44 last residues of a typical Vip3Aa sequence, render a highly unstable protein against gut fluids preventing the insecticidal function in susceptible pest species but not against the insect cell line Sf9^3^. Either the deletion or the addition of a few residues to the very C-terminal end of a Vip3A chimeric protein was found to lead to complete trypsin hydrolization of the 62 kDa fragment and to abolish the insecticidal activity^43^. A more drastic deletion of up to 220 residues in the C-terminus of Vip3Aa9 rendered a completely inactive protein whereas the deletion of the last 154 amino acids marginally decreased the toxicity against *Chilo partellus* (Lepidoptera: Crambidae) while this mutant was non-toxic against *Spodoptera litura* (Lepidoptera: Noctuidae)^55^. Experiments conducted by C-terminal sequence swapping on Vip3A proteins displayed opposite results depending on the sequence combination of the resulting chimera, including an increase in the insecticidal potency and even a broadening of the target range in comparison to the native proteins^57^. The importance of the C-terminal region in the protease resistance and toxicity is in agreement with our results clustering critical positions for the protease sensitivity and the toxicity against *S. frugiperda* in a carboxy-terminal region within the last 100 amino acids of the protein (Fig. 3).

The high divergence of the C-terminal sequences amongst Vip3A proteins has been proposed to be rather related to evolutionary diversification than to the lack of functional constraints^22^, and thus, we would expect a higher acceptance of alanine substitution in this region without hindering the protein function. Though the C-terminal region of Vip3 proteins is quite diverse, there are still several residues in the C-terminus highly conserved amongst different Vip3A sequences which are likely to pose functional or structural constraints, and therefore not likely to be subjected to positive selection pressure. This is in fact the case for all residues identified to be critical for the insecticidal performance when substituted by an alanine throughout the protein sequence (Table 3, Fig. 3). The alanine replacements in these conserved sites constitute neither a homologous nor a conservative change and, therefore, the chemical properties of the native residues may take part in crucial interactions driving insecticidal response.

The effect of alanine substitutions on the insecticidal activity of Vip3Af is similar for the two caterpillar pest species tested, with some exceptions. Substitutions T167A and E483A apparently are not as critical for *A. segetum* as for *S. frugiperda* (Table 3). In contrast, most of the selected mutations in the C-terminus of the Vip3Af (G689A, I699A, Y719A, and G727A) were more critical for the insecticidal activity against *A. segetum* than for *S. frugiperda*. Wu *et al*.^22^ proposed that Gly^689^ in the Vip3Af1(WT) (position 711 in the reference paper) was a site subjected to positive selection pressure and it is likely that this position plays its role in sequence diversification whereas the specific change to an alanine results in a negative mutation. The different behaviour of the above mentioned mutations between *A. segetum* and *S. frugiperda* supports the hypothesis of the C-terminal sequence being responsible for target diversification and specificity. It is worth noting that both pest species have quite similar susceptibility profile to Vip3A proteins and that larger differences could be observed if other species were tested (for overall different Vip3 susceptibility refer to Chakroun *et al*.^13,20^).

The only mutation rendering the proteolytic pattern “b”, W552A, suppressed the insecticidal activity. This position is located within the predicted carbohydrate binding motif (CBM 4,9), which is a common feature in all Vip3 proteins known so far with the exception of Vip3B proteins^13^. Interestingly, aromatic residues such as tryptophan, tyrosine and, less commonly, phenylalanine, are considered as key residues for CBM ligand recognition and binding^58^.

The secondary and the tertiary structure of the Vip3Af1(WT) predicted in the present work suggest that the N-terminal region is mainly composed of α-helices and the C-terminus is predominantly constituted by β-sheets, in agreement with what was described previously for Vip3 proteins^22^. This disposition might be a common feature among Vip3 proteins as judged from the high homology in their consensus sequences (Fig. 7 in Chakroun *et al*.^13^). Although a reliable conclusion on the tertiary structure cannot be drawn without further empirical information, the architecture of the Vip3Af1(WT) is predicted to have 5 domains with a high rate of disordered regions, coils and loops in the last 4 domains (Fig. 7). Furthermore, the structure predicted by the Robetta server locates the above discussed Gly^689^ (the position that gives pattern “e” when mutated to Ala) in a loop between two β-sheets just as in the model predicted for the C-terminal region by Wu *et al*.^22^.

In agreement with the *in silico* prediction, none of the cysteine substitutions in C292A, C401A or C507A affected the toxicity against *S. frugiperda*, suggesting the absence of disulphide bonds stabilizing the chain structure. Contrary to our results, the insecticidal activity of the Vip3Aa7 was seriously compromised when each native cysteine was substituted to a serine^59^; the loss of the activity of C507S was rather related to trypsin sensitivity, though the authors do not discard the involvement of a disulphide bond between the pair C^401^-C^507^. Interestingly, C^401^ is the only cysteine residue predicted to be oxidized. The role of cysteines is commonly related to protein stabilization by covalent bonding between residue pairs, although the substitution of a half-cystine can be locally compensated by either the new residue or with other amino acid residues in their vicinity, with different type of interactions other than disulphide bonds (e.g. hydrogen bonding, hydrophobic interactions, aromatic and aliphatic π interactions) yet resulting in a functional protein^60–62^. These three cysteine residues are indeed highly conserved among all Vip3A sequences described so far. The main difference between Vip3A and Vip3B proteins is a short sequence insertion rich in cysteines, along with the deletion of the Cys^507^ ^53,63^. The presence of the Cys residues could be involved in rapid evolution and diversification of Vip3 insecticidal proteins as it has been extensively described for small multimeric toxins in snake venoms^64–67^.

Despite discovery of the first Vip3 proteins more than 20 years ago, only few surveys addressing structural features implied in their mode of action are available and there is not a clear insight into their adaptive evolutionary role. The large number of Vip3 proteins and the variability of toxicity against certain closely related pest species suggests a tight relationship between structure and function. The critical positions described herein may contribute to improving the insecticidal potency and the stability of Vip3A proteins by more rational and directed molecular modifications.

## Electronic supplementary material

Supplementary information
