# Supplementary material for: Critical amino acids for the insecticidal activity of Vip3Af from Bacillus thuringiensis: Inference on structural aspects
Source: Sci Rep. 2018 May 15;8:7539. doi: 10.1038/s41598-018-25346-3 (PMC5953952; doi:10.1038/s41598-018-25346-3)
Supplement: Supplementary file 1 — Supplementary information [file 41598_2018_25346_MOESM1_ESM.pdf]

SUPPLEMENTARY INFORMATION

Critical amino acids for the insecticidal activity of Vip3Af from *Bacillus thuringiensis*. Inference on structural aspects

Banyuls, N.[1], Hernández-Rodríguez C.S. [1], Van Rie, J.[2], Ferré, J.[1 #]

Supplementary Table S1. Primers used for testing out the correct change in the mutated proteins and the results of the sequencing.

Primer	Sequence	Position† in the reference gene	Product size‡ (bp)	Vip3Af mutated protein	Codon (aa) (mutation/ wt)
wt1.fw	5’ CGATGCGATAAATACGATGCTTCATA 3’	321 - 1078	757	T167A	GCT (A)/ ACT (T)
wt1.rev	5’ ACCCAACCAATGCATGTCCT 3’			E168A	GCA (A)/ GAA (E)
				P171A	GCT (A)/ CCT (P)
				F229A	GCT (A)/ TTT (F)
				M238A	GCG (A)/ ATG (M)
				N242A	GCT (A)/ AAT (N)
				F244A	GCC (A)/ TTC (F)
				R246A	GCT (A)/ CGT (R)
				Y272A	GCT (A)/ TAT (Y)
				C292A	GCC (A)/ TGC (C)
				I301A	GCT (A)/ ATT (I)
wt2.fw	5’ CGGAGGTTATTTATGGTGATACGG 3’	1166 - 1793	627	C401A	GCT (A)/ TGT (C)
wt2.rev	5’ TGGATTACATACTCAGTTTTCGGT 3’			E483A	GCA (A)/ GAA (E)
				C507A	GCT (A)/ TGT (C)
				D519A	GCC (A)/ GAC (A)
				W552A	GCG (A)/ TGG (W)
wt3.fw	5’ AAGGACGGAGGATTTTCACAA 3’	1729 - 2281	552	G689A	GCG (A)/ GGG (G)
wt3.rev	5’ TCTACATATAATCCGGTATTATTGG 3’			I699A	GCT (A)/ ATT (I)
				L711A	GCT (A)/ CTT (L)
				Y719A	GCT (A)/ TAT (Y)
				G727A	GCT (A)/ GGA (G)
				F741A	GCT (A)/ TTT (F)

†Position at 5’ end of the forward and reverse primers for each polymerase chain reaction primer pair. Reference gene accession number AJ872070.1.
‡Using the reference gene as the template.

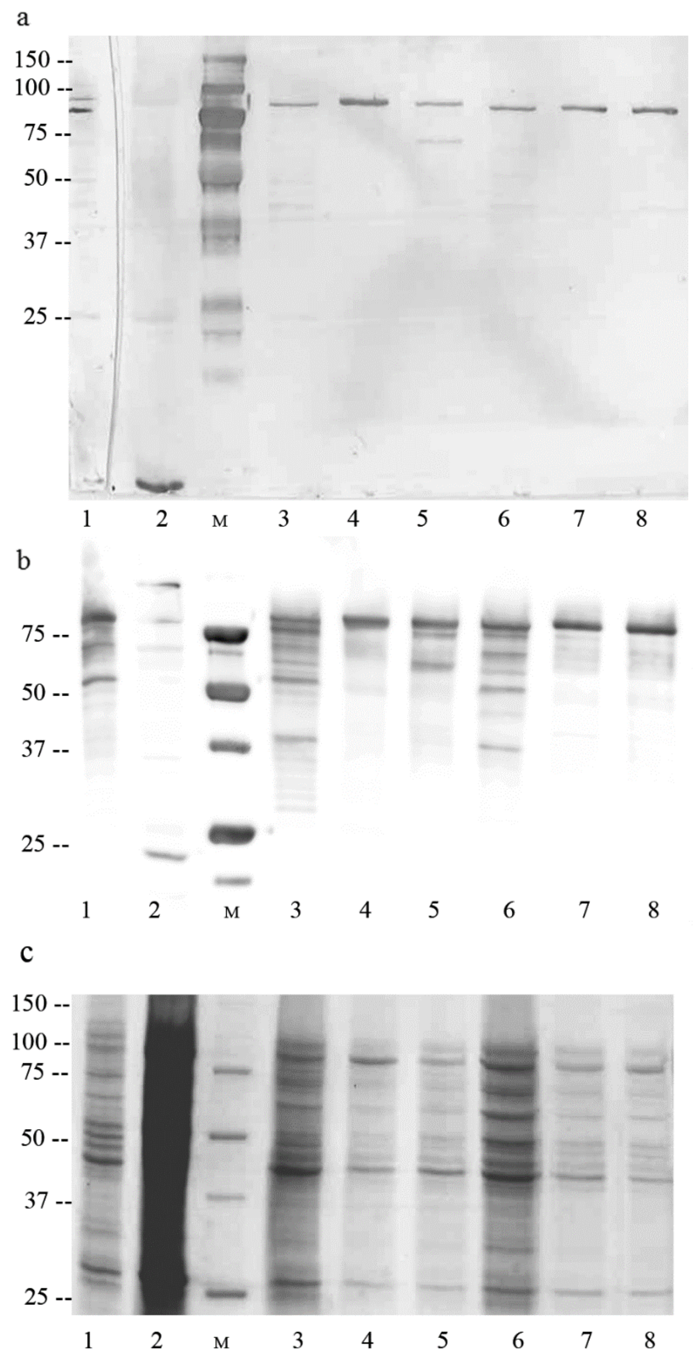

Supplementary Figure S1. Detection of Vip3Af in the crude extract after isoelectric point precipitation. Membranes were probed with (a) monocolonal antibodies against histidine for His-tag detection and (b) with polyclonal antibodies against Vip3A proteins. (c) Coomassie blue stained gel. Lane 1: Vip3Af1(WT) as a positive control; lane 2: *E. coli* wk6Ø as a negative control; lanes 3 to 8: mutants Y272A, W552A, Y719A, M238A, G689A, and E483A, respectively. “M”: molecular weight marker (kDa).

Supplementary 3D File. Three-dimensional structure of the Vip3Af1(WT) modelled *ab initio* using Robetta (confidence level of the domains conforming the model vary from 0.18 to 0.83) and representation of the selected amino acids. The structure can be visualised using PyMOL [71].
